# LncRNA CASC15 upregulates cyclin D1 by downregulating miR-365 in laryngeal squamous cell carcinoma to promote cell proliferation

**DOI:** 10.1186/s40463-022-00560-2

**Published:** 2022-02-25

**Authors:** Minhui Zhu, Caiyun Zhang, Peng Zhou, Shicai Chen, Hongliang Zheng

**Affiliations:** Department of Otorhinolaryngology Head and Neck Surgery, Changhai Hospital, Navy Military Medical University, No. 168 Changhai Road, Shanghai, 200433 People’s Republic of China

**Keywords:** Laryngeal squamous cell carcinoma, lncRNA CASC15, Cyclin D1, miR-365

## Abstract

**Background:**

This study investigated the role of lncRNA CASC15 in laryngeal squamous cell carcinoma (LSCC).

**Methods:**

This study included 58 LSCC patients. Both tumor (LSCC) and adjacent (within 3 cm around tumors) non-tumor tissues from 3 different sites of each patient were collected. CCK-8 assay was used to determine cell proliferation. The expression levels of proteins and mRNAs were determined by Western blotting analysis and qRT-PCRs, respectively.

**Results:**

CASC15 was upregulated in LSCC and high expression levels of CASC15 predicted poor survival. In LSCC tissues, CASC15 was negatively correlated with miR-365 but positively correlated with cyclin D1. In LSCC cells, overexpression of CASC15 resulted in downregulation of miR-365 and upregulation of cyclin D1. Overexpression of miR-365 did not affect the expression of CASC15 but downregulated cyclin D1. Overexpression of Cyclin D1 did not affect the expression of miR-365 and CASC15. Overexpression of CASC15 and cyclin D1 led to promoted, while overexpression of miR-365 led to inhibited LSCC cell proliferation. In addition, overexpression of miR-365 reduced the effects of overexpression of CASC15.

**Conclusion:**

Therefore, CASC15 upregulates cyclin D1 by downregulating miR-365 in LSCC to promote cell proliferation.

## Introduction

Laryngeal cancer is the most common type of head and neck cancers. The most common pathological type of laryngeal cancer is laryngeal squamous cell carcinoma (LSCC), which accounts for about 85–96% of laryngeal cancer [1]. The incidence of LSCC is not high, but this disease is considered as a major cause of cancer-related mortalities due to its rapid development, extremely aggressive nature and lack of effective therapeutic approaches [2, 3]. The overall 5-year survival rate of LSCC is still less than 50% even after active treatment, and no significant improvement in the survival of patients has been made over the past 30 years [4]. Genetic alterations are critical players in LSCC [5, 6], and characterization of novel molecular pathways involved in this disease may provide new insights into clinical treatment.

Non-coding RNAs (ncRNAs), such as long (> 200 nt) ncRNAs (lncRNAs), do not have protein-coding capacity but exert their functions as tumor suppressors or oncogenes to inhibit or promote the development of cancer [7, 8]. LncRNA-targeted therapies have also shown promising potentials for cancer treatment [9]. Currently, the clinical application of lncRNAs is limited because the functions of most lncRNAs are unknown. Therefore, characterization of the functionality of lncRNAs in cancer biology is always needed. LncRNA CASC15 has been characterized as an oncogenic lncRNA in gastric cancer and liver cancer [10, 11]. Analysis of TCGA dataset revealed the upregulation of CASC15 in head and neck cancer (0.79 vs. 0.3, LSCC is a type of head and neck cancer). Our preliminary deep sequencing data also showed that CASC15 was upregulated in LSCC and positively correlated with cyclin D1 (data not shown), which mediates cell cycle progression and has critical roles in tumor growth [12]. Our deep sequencing data also showed the inverse correlation between CASC15 and miR-365 (data not shown). It has been reported that miR-365 can directly target cyclin D1, which plays critical roles in almost all types of cancer including LSCC [13]. Therefore, we speculated that CASC15 could interact with miR-365 to regulate the expression of cyclin D1 in LSCC. In this study we investigated the involvement of cyclin D1 in LSCC and explored its interaction with cyclin D1.

## Materials and methods

### Research patients and follow-up

This study included 58 LSCC patients (36 males and 22 females, 42 to 67 years old, mean age 54.1 ± 7.3 years old) who were selected from the 134 LSCC patients admitted at Changhai Hospital, Navy Military Medical University from January 2011 to January 2014. Inclusion criteria: (1) LSCC patients who were newly diagnosed; (2) major organs were still in good condition (based on electrocardiogram, urine test, blood test, scatoscopy, X-ray and B-mode ultrasonography); (3) no previous anti-tumor therapies were initiated; (4) willing to participate in a 5-year follow-up. Exclusion criteria: (1) recurrent cases; (2) clinical disorders other than LSCC were observed; (3) history of malignancies. All patients were followed up for 5 years and patients who were lost during follow-up or died of other causes were excluded from this study. According to the stage standard established by AJCC, 10, 12, 18 and 18 cases were classified into stages I–IV, respectively. Patients’ specific clinical features were shown in Table 1.

**Table 1:** Clinical Characteristics of patients included

Characteristics	n	LncRNA CASC15 level		χ^2^ test	*P* value
		High	Low		
Total cases	58	28	30		
Gender					
Male	36	20	16	0.1534	0.8345
Female	22	8	14		
Age (years)					
≤ 60	20	8	12	0.1276	0.4532
> 60	38	20	18		
Smoking history					
Yes	30	18	12	0.8235	0.472
No	28	10	18		
HPV status					
Yes	35	19	16	0.1343	0.7823
No	23	9	14		
Clinical/pathological staging					
I + II	28	15	13	1.893	0.1566
III + IV	30	13	17		

### Ethical considerations

All the 58 LSCC patients were informed with the experimental protocols, and they all singed the informed consent. Before patient admission, this study was approved by the Ethics Committee of the Changhai Hospital, Navy Military Medical University.

### Tissues and cells

All LSCC patients were diagnosed through histopathological biopsies. Both tumor (LSCC) and adjacent (within 3 cm around tumors) non-tumor tissues from 3 different sites of each patient were collected. The weight of each tissue sample ranged from 0.1 to 0.14 g. All tissues were confirmed by 3 experienced pathologists. Human LSCC cell line UM-SCC-17A (MilliporeSigma, USA) was used. DMEM containing 10% FBS was used to cultivate UM-SCC-17A cells at 37 °C with 5% CO_2_.

### Vectors and miRNA mimic

CASC15 and cyclin D1 expression vectors were constructed by Songon (Shanghai, China) using pcDNA3.1 vectors. Negative control miRNA and miR-365 mimic were purchased from Sigma-Aldrich (USA).

### Transient cell transfections

UM-SCC-17A cells were harvested at the confluence of 70–90%. In a 6-well plate, 10^5^ cells were transfected with 10 nM CASC15 expression vector, cyclin D1 expression vector, 10 nM pcDNA3.1 vector (negative control, NC), 50 nM miR-365 mimic, or 50 nM negative control miRNA (negative control, NC) using lipofectamine 2000 transfection reagent (Sigma-Aldrich, USA). Cells without transfections were control (C) cells. Cells were harvested at 24 h after transfections to be used in the following experiments.

### RT-qPCR

UM-SCC-17A cells were harvested at 24 h after transfections and mixed with Ribozol reagent (Invitrogen, USA, 10^5^ cells with 1 ml solution) to extract total RNAs. Tissues were ground in liquid nitrogen, followed by the addition of Ribozol reagent (0.5 g tissue with 1 ml solution) to extract total RNAs. RNA samples were treated with DNase I to remove genomic DNA. RNA samples were then used as template to synthesize cDNA through reverse transcription (25 °C for 10 min, 55 °C for 20 min and 85 °C for 10 min) using AMV Reverse Transcriptase (Canvax Biotech, USA). SYBR Green Master Mix (Bio-Rad, USA) was used to prepare RT-qPCR mixtures. The expression of CASC15 and cyclin D1 were detected using 18S rRNA and GAPDH as endogenous control, respectively. Using the same amount of cells and tissues (0.5 ng tissues with 1 * 10^–6^ ml solution), miRNA extractions were performed using miRNA Isolation Kit (Geneaid, USA). After that, reverse transcriptions and RT-qPCRs were performed using All-in-One™ miRNA RT-qPCR Detection Kit* (Genecopoeia, Guangzhou, Shanghai). The expression of miR-365 was determined with U6 as the endogenous control. Three replicates were included for each experiment and 2^−ΔΔCT^ method was used to process data.

### Cell proliferation assay

DMEM containing 10% FBS (totally 10 ml) was mixed with 3 × 10^4^ UM-SCC-17A cells to prepare single cell suspensions. UM-SCC-17A cells were collected at 24 h post-transfections. The mycoplasma test of the cell line was performed, and cells were confirmed by STR analysis. A 96-well cell culture plate was used to cultivate cells at 37 °C with 5% CO_2_ in 0.1 ml cell suspension per well. Three replicate wells were set for each experiment. To detect cell proliferation, 10 μl cell counting kit-8 solution (Sigma-Aldrich, USA) was added into each well every 24 h for 4 times. After that, cells were cultivated for additional 2 h and 10 μl DMSO was added. Finally, OD values at 450 nm were measured to detect cell proliferation.

### Western blot analysis

UM-SCC-17A cells from 3 biological replicates of each experiment were collected at 24 h post-transfection, and 10^5^ cells were mixed with 1 ml RIPA solution (Beyotime, Jiangsu, China) to extract total proteins. Proteins were denatured and subjected to 12% SDS-PAGE gel electrophoresis. Following gel transfer (PVDF membrane) and blocking (in 5% non-fat milk at 25 °C for 2 h), cyclin D1 (1:1,500, ab31392, Abcam) or GAPDH (1:1,500, ab9845, Abcam) rabbit polyclonal primary antibody was added to incubate with the membranes at 4 °C overnight, followed by incubation with the secondary antibody of IgG-HRP goat anti rabbit (1:1,000, MBS435036, MyBioSource) at 25 °C for 2 h. ECL (Sigma-Aldrich, USA) was used to develop signals. The X-ray film (Sangon, Shanghai, China) was exposed for 10–60 s and Image J v1.46 software was used to process the signals.

### Statistical analyses

Mean values were calculated using the data from 3 biological replicates. Differences between 2 types of tissue were analyzed using paired t test. One-way ANOVA and Tukey test were used for the analysis of differences among multiple groups. Linear regression was used for correlation analysis. The 58 LSCC patients were divided into high (n = 28) and low (n = 30) CASC15 groups (Youden’s index) and survival curves were plotted using K-M method and were compared by log-rank test. *P* < 0.05 was statistically significant.

## Results

### CASC15 was upregulated in LSCC and predicted lower survival

**Fig. 1:**
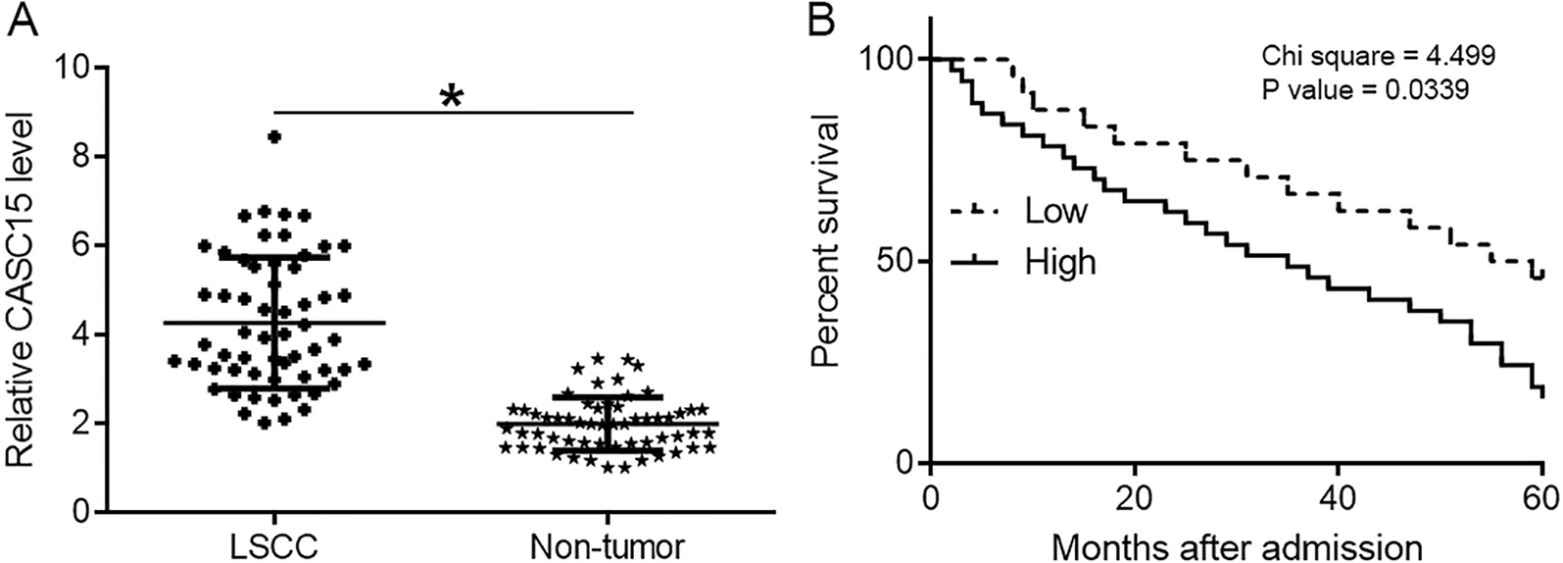
CASC15 was upregulated in LSCC and predicted poor survival. RT-qPCR analyzed by paired t test showed that the expression levels of CASC15 were significantly higher in LSCC tissues compared to that in non-tumor tissues (*, paired t test, *p* = 0.025) (**A**). Survival curve analysis showed that patients with high expression levels of CASC15 in LSCC tissues had significantly lower overall 5-year survival rate (K-M method followed by long-rank test, *p* = 0.013) (**B**)

The expression of CASC15 in two types of tissue collected from LSCC patients (n = 58) were detected by qRT-qPCR. The expression levels of CASC15 were compared between two types of tissue by performing paired t test. It showed that the expression levels of CASC15 were significantly higher in LSCC tissues compared to that in non-tumor tissues (Fig. 1A, paired t test, *p* = 0.025; LSCC tissues: 4.26 ± 1.47; non-tumor tissues: 1.99 ± 0.59). Survival curves showed that patients with high expression levels of CASC15 in LSCC tissues had significantly lower overall 5-year survival rate (Fig. 1B , K-M method followed by long-rank test, *p* = 0.013).

### CASC15 was correlated with miR-365 and cyclin D1 in LSCC

**Fig. 2:**
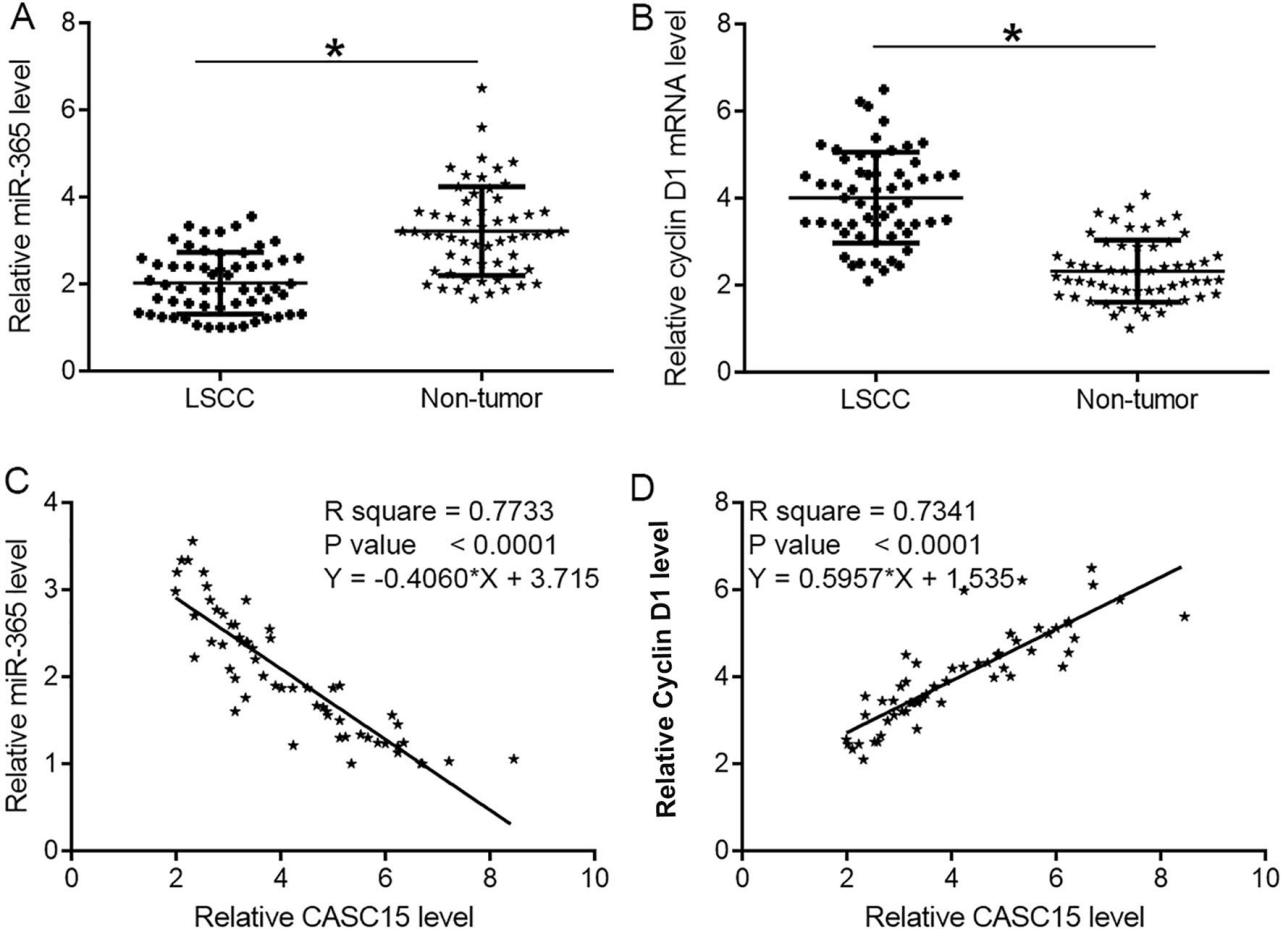
CASC15 was correlated with miR-365 and cyclin D1. The expression of miR-365 and Cyclin D1 in both types of tissue were detected by performing RT-qPCR. Paired t test analysis showed that miR-365 was significantly downregulated (paired t test, *p* = 0.015) (**A**), and cyclin D1 was significantly upregulated (paired t test, *p* = 0.043) (**B**), in LSCC tissues compared to that in non-tumor tissues. Correlations were analyzed by performing linear regression. It was observed that CASC15 was negatively correlated with miR-365 (**C**), but positively correlated with Cyclin D1 (**D**)

The expression of miR-365 and Cyclin D1 in both types of tissue were detected by RT-qPCR. Paired t test analysis showed that miR-365 was significantly downregulated (Fig. 2A, paired t test, *p* = 0.015), and cyclin D1 was significantly upregulated (Fig. 2B, paired t test, *p* = 0.043) in LSCC tissues compared to that in non-tumor tissues. Correlations were analyzed by performing linear regression. It was observed that CASC15 was negatively correlated with miR-365 (Fig. 2C, R^2^ = 0.7733, *p* < 0.0001), but positively correlated with Cyclin D1 (Fig. 2D, R^2^ = 0.7341, *p* < 0.0001) in LSCC tissues.

### CASC15 upregulated cyclin D1 by downregulating miR-365

**Fig. 3:**
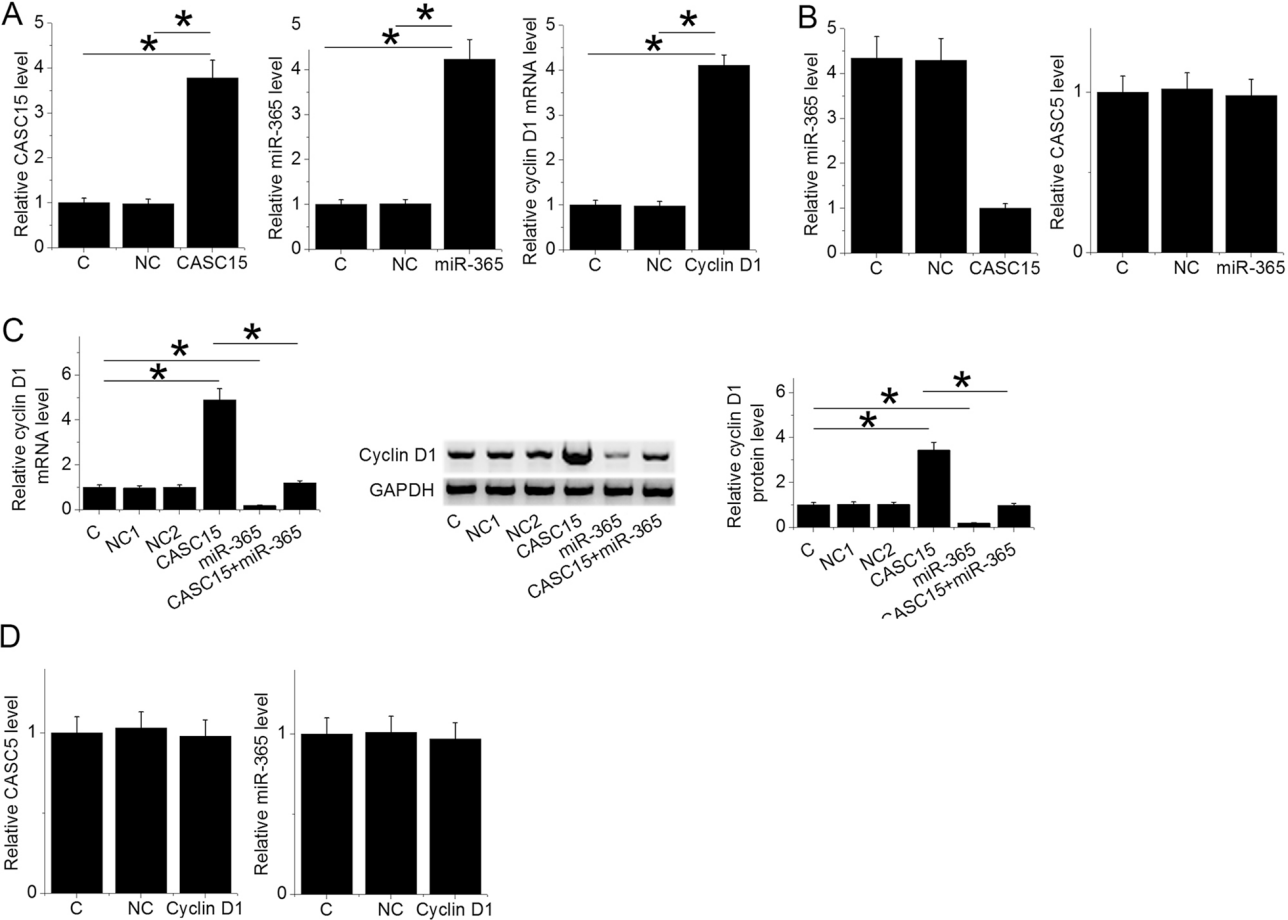
CASC15 upregulated cyclin D1 by downregulating miR-365. The expression of CASC15, miR-365 and cyclin D1 were significantly upregulated at 24 h after transfections comparing to the two controls (*, One-way ANOVA and Tukey test, *p* = 0.013) (**A**). Overexpression of CASC15 resulted in downregulation of miR-365, while overexpression of miR-365 did not affect the expression of CASC15 (*, One-way ANOVA and Tukey test, *p* = 0.024) (**B**). Overexpression of CASC15 resulted in upregulation of cyclin D1, while overexpression of miR-365 resulted in downregulation of cyclin D1 and attenuated the effects of overexpression of CASC15 (*, One-way ANOVA and Tukey test, *p* < 0.038) (**C**). Moreover, overexpression of cyclin D1 affected the expression of neither CASC15 nor miR-365 (**D**), C, Blank control group, NC1, empty vector transfection, NC2, negative control miRNA transfection, (*, *p* < 0.05)

CASC15 expression vector, miR-365 mimic and cyclin D1 expression vector were transfected into cells of LSCC cell line UM-SCC-17A. Comparing the two controls, the expression of CASC15, miR-365 and cyclin D1 were significantly upregulated at 24 h post-transfections (Fig. 3A, One-way ANOVA and Tukey test, *p* = 0.013). Comparing to the two controls, overexpression of CASC15 resulted in downregulation of miR-365, while overexpression of miR-365 did not affect the expression of CASC15 (Fig. 3B, One-way ANOVA and Tukey test, *p* = 0.024). Overexpression of CASC15 resulted in upregulation of cyclin D1, while overexpression of miR-365 resulted in downregulation of cyclin D1 and attenuated the effects of overexpression of CASC15 (Fig. 3C, One-way ANOVA and Tukey test, *p* = 0.0388). Moreover, overexpression of cyclin D1 affected the expression of neither CASC15 nor miR-365 (Fig. 3D).

### Overexpression of CASC15 resulted in accelerated LSCC cell proliferation through miR-365 and cyclin D1

**Fig. 4:**
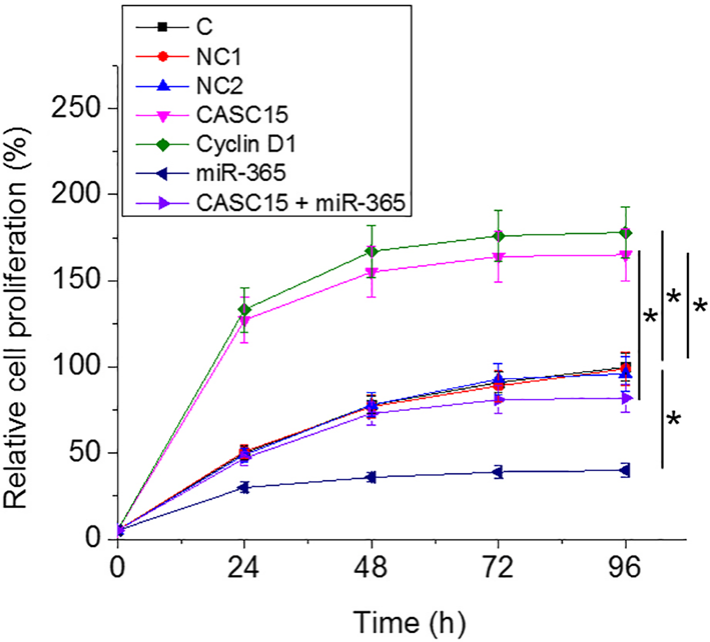
Overexpression of CASC15 resulted in accelerated LSCC cell proliferation through miR-365 and cyclin D1. Cell proliferation data analyzed by one-way ANOVA and Tukey test showed that, comparing with two controls, CASC15 and cyclin D1 overexpression led to promoted, while miR-365 overexpression led to inhibited LSCC cell proliferation, and miR-365 overexpression reduced the effects of CASC15 overexpression, C1, empty vector transfection, NC2, negative control miRNA transfection, (*, One-way ANOVA and Tukey test, *p* = 0.012)

Cell proliferation data were compared among different cell transfection groups using one-way ANOVA and Tukey test. Comparing with the two controls, overexpression of CASC15 and cyclin D1 led to promoted, while overexpression of miR-365 led to inhibited LSCC cell proliferation, and overexpression of miR-365 attenuated the effects of overexpression of CASC15 (Fig. 4, One-way ANOVA and Tukey test, *p* = 0.012).

## Discussion

The expression pattern and functions of CASC15 in LSCC were investigated in this study. We observed that CASC15 was upregulated in LSCC and CASC15 may promote the proliferation of LSCC cells by upregulating cyclin D1 though the downregulation of miR-365, which can target cyclin D1 and inhibit its function [13].

Accurate prognosis is always an important topic in cancer studies because it can be used to predict the survival of patients and guide the development of therapies to improve the survival [14]. Several prognostic markers, such as MAGE-A9 and CD44, have been developed for LSCC [15, 16], while the accuracy is still questionable. In the present study we showed that high expression levels of CASC15 were closely associated with the poor survival of LSCC patients within 5 years follow-up. However, the accuracy and efficiency remain to be further verified by large-sample-size clinical studies.

Our preliminary deep sequencing data revealed positive correlation between CASC15 and cyclin D1 (data not shown), while our RNA-IP experiments showed no direct interaction between CASC15 and cyclin D1. It was reported that cyclin D1 can be targeted by many miRNAs, such as miR-365 in colon cancer. In the present study we observed downregulated expression of cyclin D1 in LSCC cells after the overexpression of miR-365, indicating that miR-365 may also target cyclin D1 in LSCC. We showed that CASC15 is likely an upstream inhibitor of miR-365, and inhibition of miR-365 led to the upregulation of cyclin D1 and the accelerated proliferation of LSCC cells. Overexpression of miR-365 led to inhibition of LSCC cell proliferation, while overexpression of miR-365 reduced the effect of overexpression of CASC15, and there was a positive correlation between CASC15 and cyclin D1. We further identified potential targets of CASC15 to explore its contribution to LSCC. We found that transfection of miR-365 resulted in a significant decrease in CASC5-WT activity, whereas transfection of miR-365 did not cause significant changes in CASC15-MUT activity. qRT-PCR results also indicated that overexpression of miR-365 resulted in the suppression of CASC15, whereas inhibition of miR-365 resulted in increased expression levels of CASC15. These data together suggest an antagonism between miR-365 and CASC15. Therefore, a novel CASC15/miR-365/cyclin D1 pathway was characterized in the regulation of LSCC cell proliferation. However, the mechanism mediating the interaction between CASC15 and miR-365 is still unknown. LncRNAs may inhibit the function of miRNAs by serving as their sponges [17, 18], while no promising binding site of miR365 was found on CASC15 after a local blast. Therefore, mediators may exist between CASC15 and miR-365. Our future studies will try to identify those mediators.

## Conclusion

In conclusion, CASC15 was upregulated in LSCC and CASC15 may upregulate cyclin D1 by downregulating miR-365 to promote the proliferation of LSCC cells.

## Data Availability

The data that support the findings of this study are available on request from the corresponding author. The data are not publicly available due to their containing information that could compromise the privacy of research participants.
